# Ethyl *N*-(2-benzoyl-4-chloro­phen­yl)­ethane­carboximidate

**DOI:** 10.1107/S1600536812007763

**Published:** 2012-02-29

**Authors:** H. P. Sumathi, A. S. Dayananda, Grzegorz Dutkiewicz, H. S. Yathirajan, Maciej Kubicki

**Affiliations:** a Department of Studies in Chemistry, University of Mysore, Mysore 570 006, India; b Department of Chemistry, Adam Mickiewicz University, Grunwaldzka 6, 60-780 Poznań, Poland

## Abstract

In the title compound, C_17_H_16_ClNO_2_, the N=C—O—C—C fragment is planar within 0.029 (1) Å, and makes dihedral angles of 66.71 (8) and 59.61 (8)° with the planes of the chloro­phenyl and benzoyl rings, respectively. The carbonyl C=O bond is not coplanar with either of the aromatic rings; it makes angles of 42.5 and 23.5° with the normals to the ring planes. In the crystal, very weak C—H⋯O, C—H⋯Cl, C—H⋯π and π–π [inter­planar distance = 3.53 (1) Å] inter­actions are observed.

## Related literature

For background to the medical applications of benzophenones, see, for instance: Evans *et al.* (1987 ▶); Revesz *et al.* (2004 ▶); Wiesner *et al.* (2002 ▶); Zeng *et al.* (2010 ▶). A similar structure has been described by Derieg *et al.* (1970 ▶)
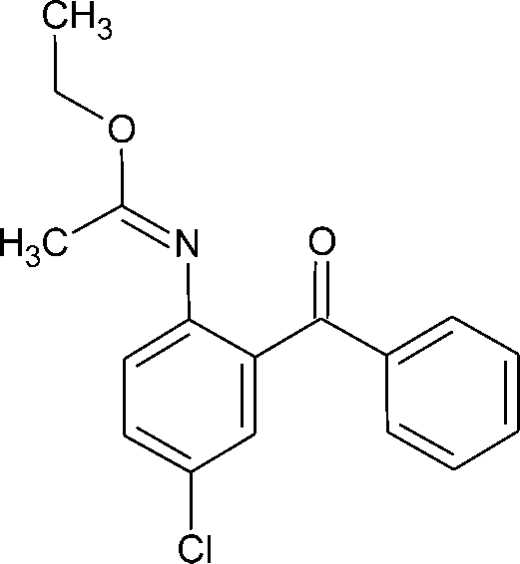


## Experimental

### 

#### Crystal data

C_17_H_16_ClNO_2_
*M* *_r_* = 301.76
Triclinic, 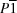

*a* = 7.9674 (11) Å
*b* = 8.6993 (17) Å
*c* = 11.596 (2) Å
α = 104.499 (17)°
β = 94.871 (14)°
γ = 95.001 (14)°
*V* = 770.4 (2) Å^3^
*Z* = 2
Mo *K*α radiation
μ = 0.25 mm^−1^
*T* = 295 K
0.35 × 0.2 × 0.15 mm

#### Data collection

Agilent Xcalibur Eos diffractometer
Absorption correction: multi-scan (*CrysAlis PRO*; Agilent, 2011 ▶) *T*_min_ = 0.991, *T*_max_ = 1.000
13278 measured reflections
3377 independent reflections
2455 reflections with *I* > 2σ(*I*)
*R*_int_ = 0.028

#### Refinement

*R*[*F* ^2^ > 2σ(*F* ^2^)] = 0.044
*wR*(*F* ^2^) = 0.117
*S* = 1.05
3377 reflections
192 parameters
H-atom parameters constrained
Δρ_max_ = 0.20 e Å^−3^
Δρ_min_ = −0.21 e Å^−3^

### 

Data collection: *CrysAlis PRO* (Agilent, 2011 ▶); cell refinement: *CrysAlis PRO*; data reduction: *CrysAlis PRO*; program(s) used to solve structure: *SIR92* (Altomare *et al.*, 1993 ▶); program(s) used to refine structure: *SHELXL97* (Sheldrick, 2008 ▶); molecular graphics: *XP* (Sheldrick, 2008 ▶); software used to prepare material for publication: *SHELXL97*.

## Supplementary Material

Crystal structure: contains datablock(s) I, global. DOI: 10.1107/S1600536812007763/nk2143sup1.cif

Structure factors: contains datablock(s) I. DOI: 10.1107/S1600536812007763/nk2143Isup2.hkl

Supplementary material file. DOI: 10.1107/S1600536812007763/nk2143Isup3.cml

Additional supplementary materials:  crystallographic information; 3D view; checkCIF report

## Figures and Tables

**Table 1:** Hydrogen-bond geometry (Å, °) *CgA* denotes the centroid of the phenyl ring C1–C6.

*D*—H⋯*A*	*D*—H	H⋯*A*	*D*⋯*A*	*D*—H⋯*A*
C5—H5⋯O22^i^	0.93	2.80	3.704 (2)	164
C6—H6⋯O14^ii^	0.93	2.73	3.648 (2)	172
C16—H16*B*⋯O22^iii^	0.96	2.66	3.614 (3)	171
C27—H27⋯Cl4^iv^	0.93	2.88	3.739 (2)	154
C25—H25⋯CgA^v^	0.93	2.90	3.744 (3)	151

Symmetry codes: (i) 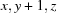; (ii) 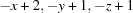; (iii) 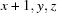; (iv) 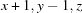; (v) 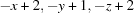.

**Fig. 1.**
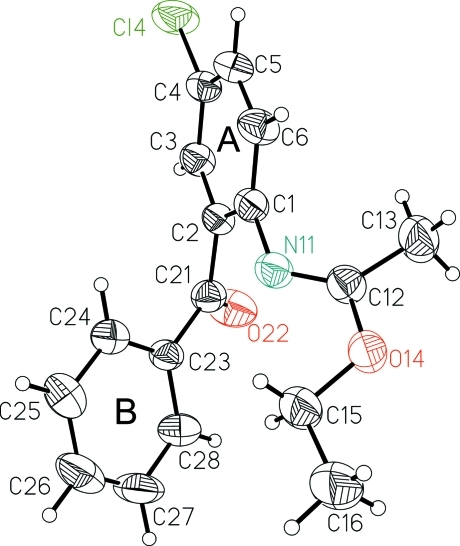
Anisotropic ellipsoid representation of 1, drawn at 50% probability level.

**Fig. 2.**
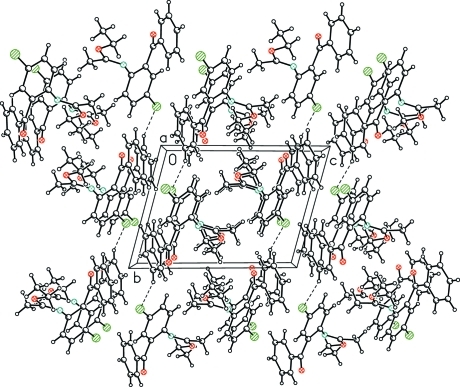
The crystal packing as seen approximately along the a-axis direction. Weak C—H···Cl hydrogen bonds are depicted as dashed lines.

## supplementary crystallographic information

### Comment

Benzophenone and related analogues have been reported to act as antiallergic, anti-inflammatory, antiasthamatic, antimalarial, anti-microbial and antianaphylactic agents (Evans *et al.*, 1987; Wiesner *et al.*, 2002). The competence of benzophenones as chemotherapeutic agents, especially as inhibitors of HIV-1 reverse transcriptase RT, cancer and inflammation, is well established and their chemistry has been studied extensively (Revesz *et al.*, 2004, Zeng *et al.*, 2010). The title compound - *N*-(2-Benzoyl-4-chloro-phenyl)-acetimidic acid ethyl ester (1, Scheme 1) - is an intermediate in the synthesis of certain anxiolytic, anticonvulsant and sedative drugs.

The conformation of *N*-(2-Benzoyl-4-chloro-phenyl)-acetimidic acid ethyl ester (1, Scheme 1) can be described by the dihedral angles between the approximately planar fragments: two aromatic rings (A and B, *cf.* Fig. 1), and the N=C—O—C—C chain (C, which is planar within 0.029 (1) Å). All these angles are close to 60°: A/B 69.14 (5) °, A/C 66.71 (8) °, B/C 59.61 (8) °. Interestingly, the C21=O22 double bond is not coplanar with either A or B phenyl rings, the C2—C21(=O22)—C23 plane makes the dihedral angle 52.81 (6) ° with the ring A and 25.51 (8) ° with ring B. Quite similar conformation was observed in the crystal structure of related compound, 2-benzoyl-4-chloroformanilide triacetylhydrazide (Derieg *et al.*, 1970).

In the crystal only some weak but directional C—H···O, C—H···Cl and C—H···π interaction can be found (*cf.* Table), and they to some extent influence the packing together with van der Waals interactions. Also the phenyl rings B from molecules related by the center of symmetry stack to some extent with the interplanar distance of *ca* 3.53 Å.

### Experimental

The title compound was obtained as a gift sample from R. L. Fine Chem., Bengaluru, India. The compound was recrystallized from dichloromethane by slow evaporation (m.p: 323 K).

### Refinement

Hydrogen atoms were put in the idealized positions, and refined as riding model. Their isotropic thermal parameters were set at 1.2 times *U*_eq_'s of appropriate carrier atoms.

### Crystal data

**Table:**

C_17_H_16_ClNO_2_	*Z* = 2
*M**_r_* = 301.76	*F*(000) = 316
Triclinic, *P*1	*D*_x_ = 1.301 Mg m^−^^3^
Hall symbol: -P 1	Mo *K*α radiation, λ = 0.7107 Å
*a* = 7.9674 (11) Å	Cell parameters from 1885 reflections
*b* = 8.6993 (17) Å	θ = 2.9–27.8°
*c* = 11.596 (2) Å	µ = 0.25 mm^−^^1^
α = 104.499 (17)°	*T* = 295 K
β = 94.871 (14)°	Block, colourless
γ = 95.001 (14)°	0.35 × 0.2 × 0.15 mm
*V* = 770.4 (2) Å^3^	

### Data collection

**Table:**

Agilent Xcalibur Eos diffractometer	3377 independent reflections
Radiation source: Enhance (Mo) X-ray Source	2455 reflections with *I* > 2σ(*I*)
Graphite monochromator	*R*_int_ = 0.028
Detector resolution: 16.1544 pixels mm^-1^	θ_max_ = 28.2°, θ_min_ = 3.0°
ω–scan	*h* = −10→10
Absorption correction: multi-scan (*CrysAlis PRO*; Agilent, 2011)	*k* = −11→11
*T*_min_ = 0.991, *T*_max_ = 1.000	*l* = −15→15
13278 measured reflections	

### Refinement

**Table:**

Refinement on *F*^2^	Primary atom site location: structure-invariant direct methods
Least-squares matrix: full	Secondary atom site location: difference Fourier map
*R*[*F*^2^ > 2σ(*F*^2^)] = 0.044	Hydrogen site location: inferred from neighbouring sites
*wR*(*F*^2^) = 0.117	H-atom parameters constrained
*S* = 1.05	*w* = 1/[σ^2^(*F*_o_^2^) + (0.0479*P*)^2^ + 0.1642*P*] where *P* = (*F*_o_^2^ + 2*F*_c_^2^)/3
3377 reflections	(Δ/σ)_max_ = 0.001
192 parameters	Δρ_max_ = 0.20 e Å^−^^3^
0 restraints	Δρ_min_ = −0.21 e Å^−^^3^

### Special details

**Table:**

Experimental. Address for R L Fine Chemicals:No 15, R L Fine Chem, KHB Industrial Area, Yelahanka New Town, Bengaluru-560 106, India. Website: http://www.rlfinechem.com
Geometry. All e.s.d.'s (except the e.s.d. in the dihedral angle between two l.s. planes) are estimated using the full covariance matrix. The cell e.s.d.'s are taken into account individually in the estimation of e.s.d.'s in distances, angles and torsion angles; correlations between e.s.d.'s in cell parameters are only used when they are defined by crystal symmetry. An approximate (isotropic) treatment of cell e.s.d.'s is used for estimating e.s.d.'s involving l.s. planes.
Refinement. Refinement of *F*^2^ against ALL reflections. The weighted *R*-factor *wR* and goodness of fit *S* are based on *F*^2^, conventional *R*-factors *R* are based on *F*, with *F* set to zero for negative *F*^2^. The threshold expression of *F*^2^ > σ(*F*^2^) is used only for calculating *R*-factors(gt) *etc*. and is not relevant to the choice of reflections for refinement. *R*-factors based on *F*^2^ are statistically about twice as large as those based on *F*, and *R*- factors based on ALL data will be even larger.

### Fractional atomic coordinates and isotropic or equivalent isotropic displacement parameters (Å^2^)

**Table:**

	*x*	*y*	*z*	*U*_iso_*/*U*_eq_	
C1	0.7628 (2)	0.41768 (19)	0.70454 (14)	0.0376 (4)	
C2	0.6659 (2)	0.33697 (19)	0.77071 (14)	0.0361 (4)	
C3	0.5286 (2)	0.4041 (2)	0.82141 (15)	0.0411 (4)	
H3	0.4601	0.3484	0.8619	0.049*	
C4	0.4940 (2)	0.5531 (2)	0.81170 (15)	0.0422 (4)	
Cl4	0.32629 (7)	0.63863 (7)	0.87998 (5)	0.0670 (2)	
C5	0.5910 (2)	0.6367 (2)	0.74955 (16)	0.0464 (4)	
H5	0.5671	0.7374	0.7439	0.056*	
C6	0.7238 (2)	0.5683 (2)	0.69612 (16)	0.0463 (4)	
H6	0.7890	0.6236	0.6535	0.056*	
N11	0.90172 (18)	0.34957 (17)	0.65447 (13)	0.0428 (4)	
C12	0.9091 (2)	0.3070 (2)	0.54356 (16)	0.0432 (4)	
C13	0.7813 (3)	0.3131 (3)	0.44432 (18)	0.0663 (6)	
H13A	0.6819	0.3528	0.4768	0.099*	
H13B	0.7517	0.2077	0.3923	0.099*	
H13C	0.8278	0.3828	0.3997	0.099*	
O14	1.04660 (16)	0.24308 (16)	0.50072 (10)	0.0514 (3)	
C15	1.1748 (3)	0.2175 (3)	0.58734 (18)	0.0611 (6)	
H15A	1.1248	0.1528	0.6353	0.073*	
H15B	1.2242	0.3191	0.6404	0.073*	
C16	1.3068 (3)	0.1356 (3)	0.5231 (2)	0.0828 (8)	
H16A	1.2558	0.0387	0.4668	0.124*	
H16B	1.3885	0.1107	0.5794	0.124*	
H16C	1.3621	0.2040	0.4810	0.124*	
C21	0.7012 (2)	0.1754 (2)	0.78588 (15)	0.0398 (4)	
O22	0.58787 (18)	0.06586 (16)	0.76157 (14)	0.0632 (4)	
C23	0.8739 (2)	0.15418 (19)	0.83424 (14)	0.0368 (4)	
C24	0.9812 (2)	0.2816 (2)	0.90603 (16)	0.0455 (4)	
H24	0.9479	0.3840	0.9217	0.055*	
C25	1.1373 (3)	0.2576 (3)	0.95454 (18)	0.0587 (5)	
H25	1.2078	0.3432	1.0049	0.070*	
C26	1.1892 (3)	0.1075 (3)	0.9288 (2)	0.0655 (6)	
H26	1.2951	0.0917	0.9613	0.079*	
C27	1.0850 (3)	−0.0190 (3)	0.8554 (2)	0.0672 (6)	
H27	1.1214	−0.1202	0.8365	0.081*	
C28	0.9271 (3)	0.0033 (2)	0.80965 (18)	0.0533 (5)	
H28	0.8554	−0.0835	0.7619	0.064*	

### Atomic displacement parameters (Å^2^)

**Table:**

	*U*^11^	*U*^22^	*U*^33^	*U*^12^	*U*^13^	*U*^23^
C1	0.0369 (9)	0.0398 (9)	0.0377 (9)	0.0072 (7)	0.0026 (7)	0.0122 (7)
C2	0.0353 (9)	0.0371 (8)	0.0365 (8)	0.0070 (7)	0.0002 (7)	0.0111 (7)
C3	0.0375 (9)	0.0466 (10)	0.0428 (9)	0.0092 (8)	0.0051 (8)	0.0165 (8)
C4	0.0411 (10)	0.0466 (10)	0.0420 (9)	0.0175 (8)	0.0043 (8)	0.0133 (8)
Cl4	0.0666 (4)	0.0787 (4)	0.0720 (4)	0.0447 (3)	0.0256 (3)	0.0314 (3)
C5	0.0540 (11)	0.0403 (9)	0.0498 (10)	0.0164 (9)	0.0026 (9)	0.0181 (8)
C6	0.0505 (11)	0.0437 (10)	0.0520 (10)	0.0090 (9)	0.0088 (9)	0.0235 (8)
N11	0.0417 (8)	0.0502 (9)	0.0418 (8)	0.0141 (7)	0.0086 (7)	0.0174 (7)
C12	0.0384 (10)	0.0510 (10)	0.0443 (10)	0.0086 (8)	0.0075 (8)	0.0175 (8)
C13	0.0553 (13)	0.0958 (17)	0.0489 (11)	0.0261 (12)	0.0009 (10)	0.0165 (11)
O14	0.0427 (7)	0.0750 (9)	0.0404 (7)	0.0197 (7)	0.0082 (6)	0.0165 (6)
C15	0.0527 (12)	0.0842 (15)	0.0502 (11)	0.0284 (11)	0.0031 (10)	0.0184 (10)
C16	0.0640 (15)	0.118 (2)	0.0739 (15)	0.0435 (15)	0.0100 (13)	0.0262 (15)
C21	0.0429 (10)	0.0360 (9)	0.0418 (9)	0.0070 (8)	0.0063 (8)	0.0112 (7)
O22	0.0490 (8)	0.0447 (7)	0.0956 (11)	−0.0023 (7)	−0.0048 (8)	0.0242 (7)
C23	0.0421 (10)	0.0353 (8)	0.0375 (9)	0.0108 (7)	0.0074 (7)	0.0145 (7)
C24	0.0478 (11)	0.0410 (9)	0.0480 (10)	0.0105 (8)	0.0025 (9)	0.0114 (8)
C25	0.0486 (12)	0.0701 (14)	0.0584 (12)	0.0067 (10)	−0.0065 (10)	0.0223 (10)
C26	0.0476 (12)	0.0906 (17)	0.0721 (14)	0.0281 (12)	0.0055 (11)	0.0404 (13)
C27	0.0729 (15)	0.0616 (13)	0.0798 (15)	0.0405 (13)	0.0148 (13)	0.0286 (12)
C28	0.0621 (13)	0.0387 (10)	0.0623 (12)	0.0166 (9)	0.0062 (10)	0.0154 (9)

### Geometric parameters (Å, º)

**Table:**

C1—C2	1.398 (2)	C15—C16	1.472 (3)
C1—C6	1.398 (2)	C15—H15A	0.9700
C1—N11	1.402 (2)	C15—H15B	0.9700
C2—C3	1.390 (2)	C16—H16A	0.9600
C2—C21	1.506 (2)	C16—H16B	0.9600
C3—C4	1.379 (2)	C16—H16C	0.9600
C3—H3	0.9300	C21—O22	1.214 (2)
C4—C5	1.380 (3)	C21—C23	1.486 (2)
C4—Cl4	1.7391 (17)	C23—C24	1.380 (2)
C5—C6	1.376 (2)	C23—C28	1.383 (2)
C5—H5	0.9300	C24—C25	1.375 (3)
C6—H6	0.9300	C24—H24	0.9300
N11—C12	1.254 (2)	C25—C26	1.373 (3)
C12—O14	1.348 (2)	C25—H25	0.9300
C12—C13	1.485 (3)	C26—C27	1.370 (3)
C13—H13A	0.9600	C26—H26	0.9300
C13—H13B	0.9600	C27—C28	1.372 (3)
C13—H13C	0.9600	C27—H27	0.9300
O14—C15	1.442 (2)	C28—H28	0.9300
			
C2—C1—C6	118.80 (15)	C16—C15—H15A	109.9
C2—C1—N11	119.15 (14)	O14—C15—H15B	109.9
C6—C1—N11	121.95 (16)	C16—C15—H15B	109.9
C3—C2—C1	119.58 (15)	H15A—C15—H15B	108.3
C3—C2—C21	118.18 (15)	C15—C16—H16A	109.5
C1—C2—C21	122.20 (15)	C15—C16—H16B	109.5
C4—C3—C2	120.03 (16)	H16A—C16—H16B	109.5
C4—C3—H3	120.0	C15—C16—H16C	109.5
C2—C3—H3	120.0	H16A—C16—H16C	109.5
C3—C4—C5	121.20 (16)	H16B—C16—H16C	109.5
C3—C4—Cl4	119.55 (14)	O22—C21—C23	120.95 (16)
C5—C4—Cl4	119.25 (13)	O22—C21—C2	119.91 (16)
C6—C5—C4	118.86 (16)	C23—C21—C2	119.12 (15)
C6—C5—H5	120.6	C24—C23—C28	119.14 (17)
C4—C5—H5	120.6	C24—C23—C21	121.28 (15)
C5—C6—C1	121.44 (17)	C28—C23—C21	119.57 (16)
C5—C6—H6	119.3	C25—C24—C23	120.16 (17)
C1—C6—H6	119.3	C25—C24—H24	119.9
C12—N11—C1	122.68 (15)	C23—C24—H24	119.9
N11—C12—O14	119.97 (16)	C26—C25—C24	120.2 (2)
N11—C12—C13	129.03 (17)	C26—C25—H25	119.9
O14—C12—C13	110.99 (15)	C24—C25—H25	119.9
C12—C13—H13A	109.5	C27—C26—C25	120.0 (2)
C12—C13—H13B	109.5	C27—C26—H26	120.0
H13A—C13—H13B	109.5	C25—C26—H26	120.0
C12—C13—H13C	109.5	C26—C27—C28	120.08 (19)
H13A—C13—H13C	109.5	C26—C27—H27	120.0
H13B—C13—H13C	109.5	C28—C27—H27	120.0
C12—O14—C15	116.82 (14)	C27—C28—C23	120.4 (2)
O14—C15—C16	108.70 (17)	C27—C28—H28	119.8
O14—C15—H15A	109.9	C23—C28—H28	119.8
			
C6—C1—C2—C3	3.2 (2)	C13—C12—O14—C15	−174.91 (18)
N11—C1—C2—C3	179.56 (15)	C12—O14—C15—C16	175.44 (19)
C6—C1—C2—C21	−179.05 (16)	C3—C2—C21—O22	50.6 (2)
N11—C1—C2—C21	−2.6 (2)	C1—C2—C21—O22	−127.22 (19)
C1—C2—C3—C4	−3.4 (2)	C3—C2—C21—C23	−127.49 (17)
C21—C2—C3—C4	178.69 (16)	C1—C2—C21—C23	54.7 (2)
C2—C3—C4—C5	1.7 (3)	O22—C21—C23—C24	−153.15 (17)
C2—C3—C4—Cl4	−177.78 (13)	C2—C21—C23—C24	24.9 (2)
C3—C4—C5—C6	0.4 (3)	O22—C21—C23—C28	25.1 (3)
Cl4—C4—C5—C6	179.81 (14)	C2—C21—C23—C28	−156.77 (16)
C4—C5—C6—C1	−0.6 (3)	C28—C23—C24—C25	−1.5 (3)
C2—C1—C6—C5	−1.2 (3)	C21—C23—C24—C25	176.78 (17)
N11—C1—C6—C5	−177.46 (16)	C23—C24—C25—C26	2.0 (3)
C2—C1—N11—C12	116.53 (19)	C24—C25—C26—C27	−0.5 (3)
C6—C1—N11—C12	−67.2 (2)	C25—C26—C27—C28	−1.5 (3)
C1—N11—C12—O14	179.59 (15)	C26—C27—C28—C23	2.0 (3)
C1—N11—C12—C13	−1.7 (3)	C24—C23—C28—C27	−0.5 (3)
N11—C12—O14—C15	4.1 (3)	C21—C23—C28—C27	−178.80 (18)

### Hydrogen-bond geometry (Å, º)

CgA denotes the centroid of the phenyl ring C1 - C6

**Table:**

*D*—H···*A*	*D*—H	H···*A*	*D*···*A*	*D*—H···*A*
C5—H5···O22^i^	0.93	2.80	3.704 (2)	164
C6—H6···O14^ii^	0.93	2.73	3.648 (2)	172
C16—H16*B*···O22^iii^	0.96	2.66	3.614 (3)	171
C27—H27···Cl4^iv^	0.93	2.88	3.739 (2)	154
C25—H25···CgA^v^	0.93	2.90	3.744 (3)	151

Symmetry codes: (i) *x*, *y*+1, *z*; (ii) −*x*+2, −*y*+1, −*z*+1; (iii) *x*+1, *y*, *z*; (iv) *x*+1, *y*−1, *z*; (v) −*x*+2, −*y*+1, −*z*+2.
